# Prevalence of anemia among school-age children in Ethiopia: a systematic review and meta-analysis

**DOI:** 10.1186/s13643-018-0741-6

**Published:** 2018-05-24

**Authors:** Robel Tezera, Zekariyas Sahile, Delelegn Yilma, Equilnet Misganaw, Ermiyas Mulu

**Affiliations:** 1 Department of Medical Radiological Technology, Division of Public Health, School of Medicine, Addis Ababa University, Addis Ababa, Ethiopia; 2 Department of Public Health, College of Health Science and Medicine, Ambo University, Ambo, Ethiopia; 3 Human Resources for Health (HRH) Department, JHPIEGO/Ethiopia, Addis Ababa, Ethiopia

**Keywords:** Prevalence, Anemia, Iron-deficiency anemia, School children, Ethiopia

## Abstract

**Background:**

Anemia continued to become a major public health problem in developing nations including Ethiopia. Especially, school children are more vulnerable for anemia and consequences of anemia. Generating accurate epidemiological data on anemia in school children is an important step for health policy maker. There are limited evidences on anemia prevalence in school-age children in Ethiopia. This study aimed to synthesize the pooled prevalence of anemia in school-age children in Ethiopia.

**Methods:**

This systematic review and meta-analysis was followed the PRISMA guidelines. Comprehensive searched was conducted in PubMed/MEDLINE, Cochrane Library, Google Scholar, HINARI, and Ethiopian Journal of Health Development for studies published before 2016, supplemented by manual searches to identify relevant studies. Two review authors independently selected studies, extracted data, and assessed quality of studies. The Cochrane *Q* test and *I*^2^ test statistic were used to test heterogeneity through studies. The overall prevalence was calculated using random-effects model of DerSimonian–Laird method.

**Results:**

From 831 obtained studies, 13 articles included in the meta-analysis. The pooled prevalence of anemia among school children in Ethiopia was 23% (95% CI 18–28%). The prevalence of anemia in male and female school-age children was 27% (95% CI 20 and 34%) and 24% (95% CI 18 and 30%), respectively.

**Conclusions:**

This study found that prevalence of anemia was a moderate public health problem in school children. Due to the complications of anemia for school children, preventative planning and control of anemia among school children in Ethiopia is necessary.

**Electronic supplementary material:**

The online version of this article (10.1186/s13643-018-0741-6) contains supplementary material, which is available to authorized users.

## Background

Anemia is a public health problem both in developed and developing countries, including Ethiopia [1]. The causes of anemia are multifactorial [1–4]. Iron deficiency is the primary cause of anemia which results iron-deficiency anemia (IDA). However, it also coexists with malaria and parasitic infection [1, 2]. Iron is an essential micronutrient and major cause of anemia, intrinsically found in every cells of human body and has several metabolic function including hemoglobin transport and storage, DNA synthesis, electron transport, and energy production [2–4].

According to the World Health Organization (WHO) report, anemia is the most common hematologic manifestation. Globally, around 1.62 billion people are affected by anemia that accounts more than 24.8% of the world population and from 30 to 50% of anemia was caused due to iron deficiency [1]. Iron-deficiency anemia (IDA) resulted 273,000 deaths in the world, and 97% of deaths were occurred in developing countries [5].

Studies have documented that rapid physical and physiological development makes school-age children more vulnerable for anemia, especially for IDA [1, 6, 7]. Based on WHO report, anemia affects 45.7 to 49.1% of school-age children in the world and prevalence of anemia among school-age children in Africa ranged from 64.3 to 71% [1]. Consequences of anemia on school-age children are poor psychomotor development, negative last-longing effects on central nervous system [8], poor IQ, poor school performance [9], reduced work capacity, and poor quality of life [3, 10]. The economic impact is also significant; annually, more than US$450 million loses in gross domestic product due to vitamin and mineral deficiencies in Ethiopia. However, scaling up micronutrient interventions would cost less than US$51 million per year [11].

The risk factors for anemia are multifaceted including malaria, renal disease, and nutritional deficiency [12, 13]. Studies also showed that schistosomiasis infection, hookworm infection, inherited disorders [13, 14], diarrhea, and fever in 6–59-month children [15] are associated with risk of developing anemia. Socio-economic factors like poverty, poor sanitation, low income, monotonous diet, parent’s level of education, and community factors are also related with prevalence of anemia [13].

In Ethiopia, few evidences are available regarding the national magnitude of anemia among pre-school children and pregnant women. Ethiopia 2016 Demography Health Survey indicated that 56% of 6–59-month-old children were anemic [16]. There is limited recent information on national prevalence of anemia in school-age children in Ethiopia. National survey was conducted on prevalence of anemia among grade 3 and 4 children in 2000. But, the evidence was not recent, and data was collected only from grade 3 and 4 students. Hence, due to absence of recent and comprehensive systemic review about prevalence of anemia in school-age children in Ethiopia, we conducted meta-analysis in order to understand and explain the differences in various studies with age, sex, residence, and study period. Thus, the aim of this study is to review evidences regarding prevalence of anemia among school-age children in Ethiopia.

## Methods

### Search strategy

This systematic review and meta-analysis was performed according to the Preferred Reporting Items for Systematic review and Meta-Analysis (PRISMA) guidelines [17] (see Additional file 1: Table S1). We conducted extensive search in PubMed/MEDLINE, Cochrane Library, Google Scholar, HINARI, and Ethiopian Journal of Health Development for studies published before 2016. It was supplemented by manual searches to identify relevant unpublished studies. Search terms used included “Anemia” or “Hemoglobin,” “school children,” and “Ethiopia.” We also screened reference list of included studies. This systematic review and meta-analysis was not registered with PROSPERO.

### Selection criteria

All population-based studies which reported the prevalence of anemia among school children in Ethiopia using English language were included. The main outcome of interest was prevalence of anemia using the WHO criterion for anemia [4]. Ethiopian school-age children were considered as study population (5–17 years of age). Studies were excluded if they were not primary studies (such as review articles, conference abstract, editorials).

### Data extraction and quality assessment

Two authors independently reviewed titles and abstract of included articles and reviewed full-text of the selected articles according to the eligibility criteria. Moreover, discrepancies between authors were resolved through discussion and consensus.

Data extractions were conducted by two authors independently. We resolved disagreement by verification and further discussion. The following data were extracted for analysis: author, publication year, survey period, setting of the study, sex, sample size, sample selection methods, number of children with anemia, and type of diagnostic criteria.

Quality assessment was conducted by two experts based on Hoy 2012 tool using 10 criteria addressing internal and external validity [18]. The items included (1) representation of the population, (2) sampling frame, (3) methods of participants’ selection, (4) non-response bias, (5) data collection directly from subjects, (6) acceptability of case definition, (7) reliability and validity of study tool, (8) mode of data collection, (9) length of prevalence period, and (10) appropriateness of numerator and denominator. Each item was assessed as either low or high risk of bias. Unclear was regarded as high risk of bias. The overall risk of bias was then scored according to the number of high risk of bias per study: low (≤ 2), moderate (3–4), and high (≥ 5) (see Additional file 2: Table S2).

### Operationalization of variable

The WHO criteria were used to determine hemoglobin (Hgb) cutoff point for anemia. Hgb levels lower than 11.5 and 12 g/dl were considered as anemic for age ranges from 5 to 11 and 12–15 years old. Hgb level below 13 g/dl was considered anemic for boys above 15 years of age [4].

### Statistical analysis and synthesis

The statistical software Review Manager (RevMan) 5.3 was used for data analysis. The variance of anemia prevalence in each article was computed based on the binomial distribution formula by extracting the frequency sample size from published data [19]. Findings are illustrated in the form of forest plots and tables. Having used heterogeneity test, Cochran *Q* (*P* value of less than 0.10 considered to be significant) and *I*^2^ statistics (at least 50% considered to be significant) [20], we found significant variations between the study findings. Therefore, we used a random-effects model with 95% confidence interval (CI) for the estimations. Subgroup analysis was performed based on quality of included studies, sexes, urban/rural setting, and survey period.

Funnel plots analysis, Egger weighted regression and Begg rank correlation test were done to detect publication bias (*P* < 0.05 was considered as suggestive of statistically significant publication bias) [20, 21].

## Results

### Identified studies

**Fig. 1:**
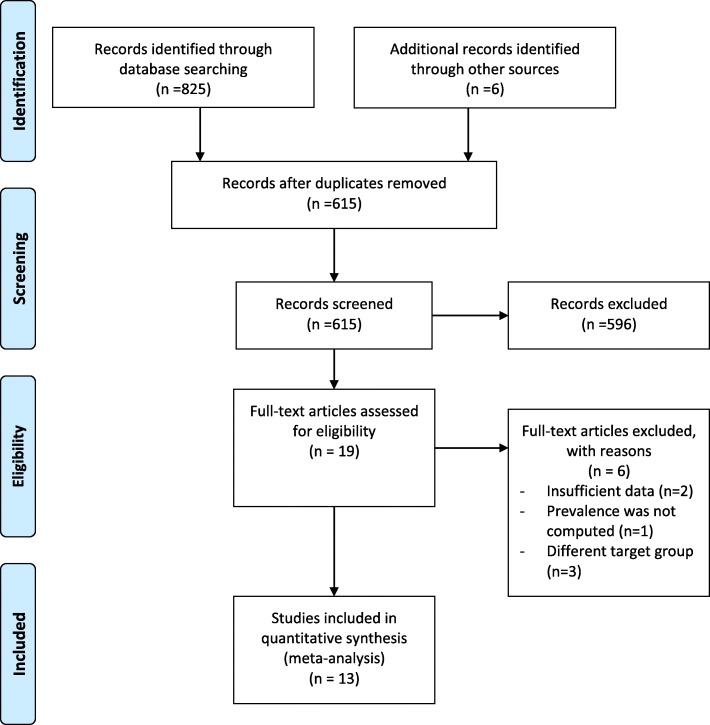
PRISMA flow chart diagram describing selection of studies for systematic review and meta-analysis on prevalence of anemia among school-age children in Ethiopia

A total of 831 articles were retrieved by literature search (Fig. 1). Of these, 216 were excluded because of duplication, 596 did not relate to the aim of this meta-analysis, 6 did not meet eligibility criteria, and 13 were included in the meta-analysis.

### Description of included studies

Twelve full-text articles and one abstract pertaining to the original articles were included in this systematic review and meta-analysis. Out 13 articles, 6 were conducted in the community setting [22–27]; remaining 7 articles were conducted in school setting [2, 6, 28–32]. The earliest study was conducted before 1993, but the exact period was not reported [32] and the latest was in 2016 [24]. All articles followed cross-sectional study design. Study population varied from 355 [23] to 14,740 [32] school-age children, between 5 and 19 years of old. A total of 22,104 school children were included in the study (54.7% were male and 45.3% females). Overall information regarding prevalence of anemia were obtained from eight regions of Ethiopia: Addis Ababa [31, 32], Amhara [26, 33], Oromia [22, 27], Tigray [30], Southern region [6, 24], Somali [23], Harar [25, 29], and Afar [2] (Table 1).

**Table 1:** Characteristic of included studies in systematic review and meta-analysis

Author	Survey period	Place of the study	Setting	Sample size	Sampling procedure	Age category (years)	Anemia (gender subgroup)		Overall prevalence (%)
							Male (%)	Female (%)	
Wolde-Gebriel et al. [32] 1993	Not reported	Shoa region/Addis Ababa	Facility-based	14,740	Simple random sampling	6–18	21	15	18.6
Mekasha and Zerfu [31] 2009	2003	Addis Ababa	Facility-based	707	Random cluster sampling	–	–	–	5.83
Herrador et al. [26] 2014	2009	Libo Kemkem and Fogera/Amhara	Community-based	764	Multistage cluster sampling	4–15	–	–	30.9
Alelign et al. [33] 2015	2010	Durbeta town/Amhara	Facility (school)	384	Multistage random sampling	5–15	10	11	10.7
Mahmud et al. [30] 2013	2010	Tigray	Facility-based	525	Systematic random sampling	6–15	15	7	11
Assefa et al. [27] 2014	2011	Jimma Town/Oromia	Community-based	404	Systematic random sampling technique	6–14	41	35	37.6
Mesfin et al. [29] 2015	2012	Kersa/Harar	Facility-based	1755	Simple Random sampling	5–14	27	27	27.1
Teji et al. [25] 2016	2012–2013	Babile district/Harar	Community-based	547	Simple random sampling	10–19	–	32	32
Desalegn et al. [22] 2104	2013	Jimma Town/Oromia	Community-based	586	Multistage randomsampling	6–12	40	36	43.7
Gutema et al. [23] 2104	2013	Filtu Town/Somali region,	Community-based	355	Systematic Random Sampling.	5–15	28	19	23.66
Adem et al. [2] 2015	2014	Berahle district/Afar	Facility-based	338	Multi stage Random sampling	14–19	–	23	22.8
Tesfaye et al. [6] 2015	2014	Bonga Town/ Southern region	Facility-based	408	Systematic random sampling	12–19	9	19	15.2
Chane et al. [24] 2016	2016	Mihur aklil district, Gurage Zone/Southern	Community-based	517	Systematic simple random sampling	5–10	58	42	21.71

### Risk of bias and heterogeneity

Quality assessment was conducted for each study in ten different items using the risk of bias tool [18]. Of the 13 included studies, our summary assessment was low risk of bias for seven studies (53.8%) [6, 22, 23, 25, 29, 30, 33], moderate risk of bias for four studies (30.8%) [2, 24, 26, 27], and high risk of bias for two studies (15.4%) [31, 32].

The included studies exhibited high heterogeneity according to Cochrane *Q* test (*Q* test *p* = 0.00001) and *I*^2^ test (98%), which is indicative to using random-effects model. However, the Egger weighted regression statistics (*p* < 0.05) and Begg rank correlation statistics (*p* = 0.06) indicated no evidence of publication bias. There was no sign of publication bias and asymmetry in the funnel plot (see Additional file 3: Figure S3).

To reduce the heterogeneity, subgroup analysis was performed based on the quality of included studies, enrolment date of study, sexes, and urban/rural setting. Heterogeneity in rural studies was 78.0% and studies conducted from 2003 to 2011 year were 99.0% (Table 2). Nonetheless, the heterogeneity in all subgroups was considerable.

**Table 2:** Subgroup analysis of the prevalence of anemia by risk of bias, sex, urban/rural setting, and enrolment date of study using chi^2^ test for heterogeneity

	Prevalence	95% CI	*P* value	*I*^2^ (%)
Risk of bias				
High risk	0.13	0.00, 0.25	0.01	99.0
Moderate risk	0.28	0.22, 0.34	0.00	95.0
Low risk	0.23	0.15, 0.32	0.01	98.0
Sex				
Male	0.27	0.20, 0.34	0.01	97.0
Female	0.24	0.18, 0.30	0.01	97.0
Enrolment date of study				
2003–2011	0.19	0.09, 0.30	0.01	99.0
2012–2016	0.27	0.20, 0.33	0.01	95.0
Rural/urban setting				
Rural	0.33	0.26, 0.40	0.00	78.0
Urban	0.24	0.10, 0.37	0.03	98.0

### Prevalence of anemia

**Fig. 2:**
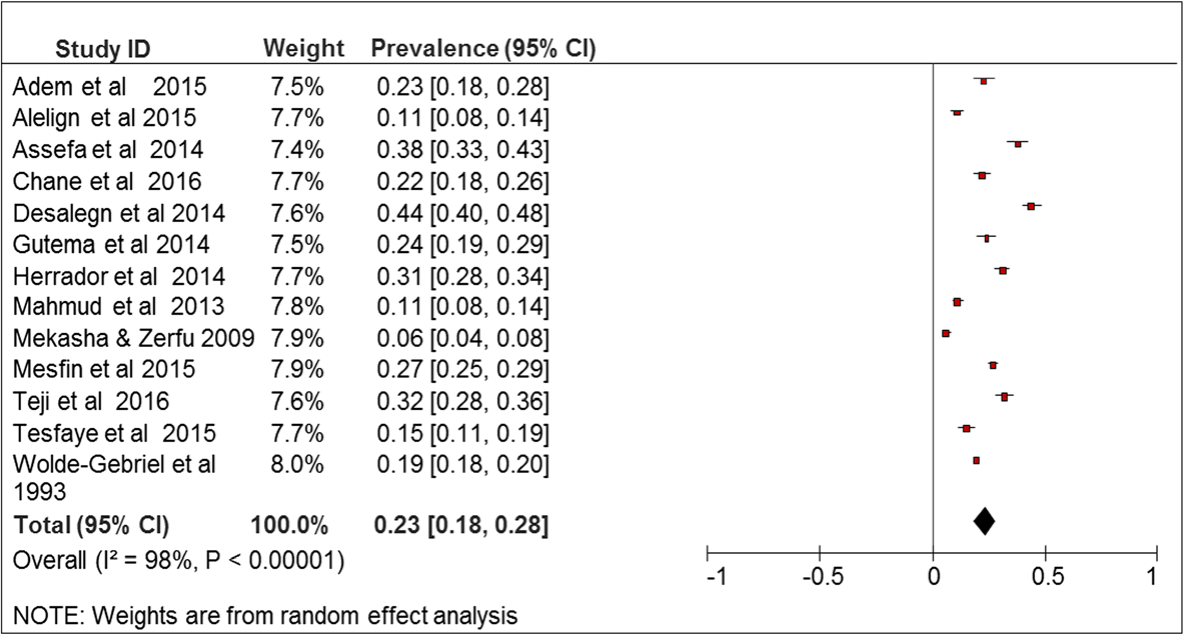
Forest plot of 13 studies on prevalence of anemia among school-age children in Ethiopia, 2003–2016

The prevalence of anemia among school-age children in Ethiopia varies from 5.83% of 707 school children in Addis Ababa [31] and 43.7% of 586 school children in Jimma town, southern region of Ethiopia [22]. The overall prevalence of the meta-analysis of 13 studies, according to the Der Simonian-Laird random-effects model, revealed that the pooled prevalence of anemia among school-age children in Ethiopia was 23% (95% CI 18–28%) (Fig. 2). Meta-analysis of eight articles on severity of anemia revealed that, among anemic children, 61% (*I*^2^ = 99%), 21% (*I*^2^ = 99%), and 11% (*I*^2^ = 98%) had mild, moderate, and severe anemia, respectively.

Subgroup analyses showed that the prevalence of anemia among male school-age children in Ethiopia was 27% (95% CI 20%, 34%) and the prevalence of anemia among female school-age children was 24% (95% CI 18%, 30%) which was estimated using 11 studies. Majority of the articles reported high prevalence in younger age category (5–9 years) [6, 12, 24, 29, 34]. Based on risk of bias, the 13 included studies were categorized into three: high risk of bias (16.3%), moderate risk (30.6%), and low risk (53.1%). The prevalence of anemia in moderate risk studies (28%; 95% CI 22 and 34%) was higher than studies with high risk of bias (13%; 95% CI 0 and 25%).

According to enrolment date of study, the 12 included studies were divided into two categories: studies conducted from 2003 to 2011 (58.2%) and studies conducted from 2012 to 2016 (41.8%). Prevalence of anemia was higher into the recent studies. According to residence, 6 included studies were divided into two categories: studies conducted in rural setting (20.75) and studies conducted in urban setting (79.25). The prevalence of anemia in rural setting (33%; 95% CI 29 and 36%) was higher than studies conducted in urban setting (21%; 95% CI 20 and 23%) (Table 2).

## Discussion

The prevalence of anemia was varying from 5.83 to 43.7%. The highest prevalence of anemia was reported in 2013 in Southwest Ethiopia (Jimma) [22]. The smallest prevalence was reported in 2009 among school children in Addis Ababa, Ethiopia [31]. In this study, we tried to estimate the overall prevalence of anemia among school going children in Ethiopia by reviewing the findings of available studies. The overall prevalence of anemia was 23%. The result was higher from the national survey conducted in Ethiopia in 2000. The reason could be target population, geographical, and sample size variations. The national survey which was conducted in 2000 was included only grade 3 and 4 children. The 2000 national survey was conducted in 11 regions, and this meta-analysis was based on data form eight regions of Ethiopia. Our estimated pooled prevalence of anemia (23%) was consistent with 2016 national survey (25.8%) [35]; nonetheless, there were little age variations.

According to WHO, anemia is a public health problem only when the prevalence exceeds 5% of the population. The WHO classification for mild, moderate, and severe is when its prevalence exceeds 5, 20, or 40%, respectively [36]. Thus, the meta-analysis revealed moderate prevalence of anemia among school going children in Ethiopia. According to the WHO definition, five articles reported mild prevalence of anemia, seven articles reported moderate prevalence of anemia, and only one article reported severe prevalence of anemia. According to this review, anemia was a major public health problem for all population included in this study.

The result was similar with systematic review conducted in Africa (Some of the included countries are South Africa, Nigeria, Cote d’Ivoire, Uganda, Rwanda, Kenya, Botswana, and Burkina Faso). The systematic review reported that the majority of the articles reported moderate prevalence of anemia [34]. However, it was a little bit a higher from the review conducted in South Africa. The highest prevalence reported in South Africa was 22% [37]. The reason could be geographical and socio-economic variations between the two countries.

In this study, male school-aged children had higher prevalence of anemia (28%) compared with female counterparts (25%). Though, there was no significant difference between the gender subgroups (*P* = 0.42). The potential explanation could be sample size, geographical, and research setting variations between included studies.

According to this review, anemia was a major public health problem for all population included in this study. The prevalence of anemia in Ethiopia depends on numerous factors such as socio-economic status, Hookworm and intestinal parasite infection, poor nutritional status, and inadequate food consumption [13, 36, 38]. A meta-analysis report shows that iron supplementation improves hematologic outcomes among primary school-aged children in low- or middle-income settings [39]. In addition, national fortification programs of micro-nutrients my help to reduce prevalence of anemia. Even with adequate iron intake, bioavailability of iron may reduce due to little consumption fruits and foods of animal region.

### Strengths and limitations

The extensive searches using different database and different searching strategy (manual and electronic) were the strength of this study. Data extraction was also conducted using pre-determined tool and was extracted by two authors independently to minimize bias. The study also reviewed potential risk factors for anemia. Quality assessment was also conducted by two independent authors, and all the included studies had moderate and high quality.

There are potential limitations to this study. The data were obtained from eight regions of Ethiopia, which comprises around 2.5 million target population. However, the analyzed pooled prevalence may not fully represent the prevalence of anemia in Ethiopia because there is lack of evidences in some parts of the country. Only Hb measurement and WHO cutoff point was used to determine status of anemia. Because of small sample size, meta-regression was not conducted to identify statistical deference between different regions of Ethiopia. Meta-analysis was also conducted by excluding poor research setting; however, the result (25%) was not significantly different from the overall prevalence (*P* = 0.36).

High heterogeneity was recorded (large *I*^2^ and small *P* value) among included studies. The source of high heterogeneity could be because the studies were conducted in different regions of Ethiopia. High heterogeneity was addressed by using random-effects model to compute pool prevalence. Random-effects model considers any heterogeneity embedded in meta-analysis.

## Conclusion

This systematic review and meta-analysis revealed moderate prevalence of anemia among school-age children in Ethiopia. Thus, adequate intervention should be designed by policy makers, health care community, and researcher to alleviate the problem. Further studies should be conducted to accurately determine potential risk factors for high prevalence of anemia.

## Additional files

Additional file 1: **Table S1.** PRISMA Checklist. (PDF 203 kb)

Additional file 2: **Table S2.** Risk of bias assessment of included studies using the Hoy 2012 tool. (XLSX 12 kb)

Additional file 3: **Figure S3.** Funnel plot of 13 studies on prevalence of anemia among school-age children in Ethiopia, 2003–2016. (WMF 2 kb)

## Abbreviations

CI: Confidence interval
Hgb: Hemoglobin
IDA: Iron deficiency anemia
PRISMA: Preferred Reporting Items for Systematic Review and Meta-Analysis
RevMan: Review manager
WHO: World Health Organization (WHO)
